# Bubble Hair and the Usefulness of Trichoscopy

**DOI:** 10.5826/dpc.1004a81

**Published:** 2020-10-26

**Authors:** Aurora Alessandrini, Michela Starace, Francesca Bruni, Bianca Maria Piraccini

**Affiliations:** 1 Department of Experimental, Diagnostic and Specialty Medicine, Division of Dermatology, University of Bologna, Italy

**Keywords:** bubble hair, hair ironing, hair shaft, hair fragility, hair disorders

## Introduction

Bubble hair abnormality describes hair damage characterized by the formation of air cavities within the cortex of the hair, due to the high temperature of water inside the shaft, that induces keratin hydrolysis and local air expansion. It is linked to excessive cosmetic treatments, in particular, prolonged exposure of damp hair to the high temperatures (> 125 C°) of blow dryers or electric curlers, and is typically encountered in women. We describe the trichoscopic features of 3 cases of bubble hair abnormality highlighting the utility of trichoscopy.

## Case Presentation

A 51-year-old Caucasian woman complained of a 6-month history of hair breakage that started with frequent at-home hair dyeing. Physical examination revealed a circumscribed area of short and broken hairs localized to her vertex. In-vivo trichoscopy revealed numerous hair shafts containing irregularly spaced bubbles and broken hairs. We made a diagnosis of bubble hair abnormality and advised her to discontinue all dye and heat-related practices.

**Figure 1:**
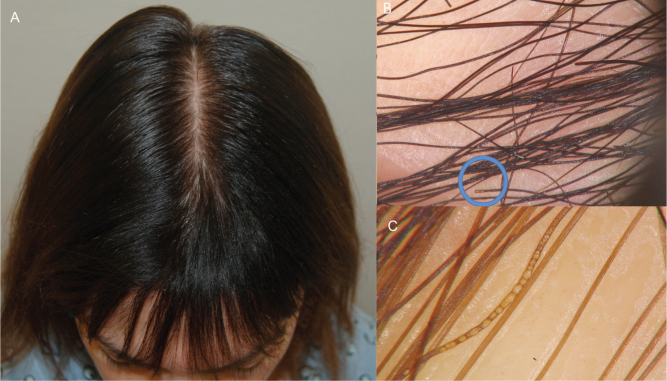
(A) Clinical picture of a female patient affected by bubble hair abnormality in the anterior region of the fringe. (B) Clear evidence of bubble hair with trichoscopy at ×20 magnification, blue circle. (C) A broken hair and a bubble hair at ×70 magnification; FotoFinder Systems.

A 42-year-old Caucasian woman complained of increased hair shedding. Trichoscopy showed signs of telogen effluvium, and upon careful observation of the anterior region of the fringe, some bubble hairs were discovered (Figure 1). The patient admitted to wrapping the hair of the frontal hairline over a round metal brush for several minutes while blow drying it.

A 35-year-old Caucasian woman complained of diffuse hair loss and the presence of damaged strands of hair that resulted after hot-combing the hair while it was still wet. A tuft of short broken hairs, paler than the surrounding hair, was evident in the affected area. Trichoscopy showed the presence of broken and bubble hair in the site of the thermal trauma.

## Conclusions

Cosmetic hair treatments, such as hair straighteners and relaxers, cleave disulfide bonds and can cause hair damage to the superficial cuticles. The loss of lipids on the hair surface during chemical hair treatments may lead to loss of cohesiveness between cuticle cells, and the reduction in the hair shaft cysteine levels correlates to a greater susceptibility to hair weathering and hair fragility. For a correct diagnosis in cases of hair fragility, direct examination of hair shafts with trichoscopy is recommended.

In the literature, previous reports have underlined the utility of trichoscopy for bubble hair abnormality and describe white oval spaces with Swiss-cheese structure and dysmorphia of the distal hair shaft [1,2].

In 2 of our patients, there was a history of blow drying wet hair, while 1 patient had only bleached it with an at-home procedure. When wet, the hair fiber can expand up to 30% in diameter and it retains water within its structure, resulting in the formation of expanding and destructive bubbles within the fiber. As reported in Case 1, chemical treatments and previous hair damage may also precipitate the onset of bubble hair abnormality and lower the threshold for bubble formation [2].

All our patients improved after discontinuation of hot blow drying or avoiding the application of chemicals, confirming the acquired nature of the disease.

In conclusion, bubble hair abnormality is an acquired hair shaft disorder that can present as a circumscribed area of broken hairs that do not grow, especially when there is a history of frequent cosmetic or thermal hair practices.
